# Enhancement of the effects of intermittent parathyroid hormone (1-34) by bone morphogenetic protein in a rat femoral open fracture model

**DOI:** 10.1186/s13018-019-1470-9

**Published:** 2019-11-29

**Authors:** Shozo Kanezaki, Masashi Miyazaki, Toshinobu Ishihara, Naoki Notani, Tetsutaro Abe, Yuhta Tsubouchi, Masashi Kataoka, Hiroshi Tsumura

**Affiliations:** 1 Department of Orthopaedic Surgery, Faculty of Medicine, Oita University, Yufu-shi, Oita, 879-5593 Japan; 2 Advanced Trauma, Emergency, and Critical Care Center, Oita University Hospital, Oita, Japan; 3 Department of Rehabilitation, Oita University Hospital, Oita, Japan; 4 Physical Therapy Course of Study, Faculty of Welfare and Health Sciences, Oita University, Oita, Japan

**Keywords:** Parathyroid hormone, Teriparatide, Bone morphogenetic protein, Open fracture, Femur

## Abstract

**Background:**

Nonunion in cases of open fracture is common. Both bone morphogenetic protein 2 (BMP-2) and parathyroid hormone (PTH) have been used to enhance bone healing. We investigated the combination of BMP-2 and PTH and examined the effects on a rat model of open femoral fractures.

**Methods:**

Group I (*n* = 11) was implanted with control carrier. Group II (*n* = 12) was implanted with carrier containing 1 μg of recombinant human BMP-2 (rhBMP-2). Group III (*n* = 12) was implanted with carrier alone, followed by injections of PTH 1-34. Group IV (*n* = 11) was implanted with carrier containing 1 μg of rhBMP-2, followed by injections of PTH 1-34. Group V (*n* = 11) was implanted with carrier containing 10 μg of rhBMP-2. Group VI (*n* = 11) was implanted with carrier containing 10 μg of rhBMP-2, followed by injections of PTH 1-34. Rats were euthanized after 8 weeks, and their fractured femurs were explanted and assessed by manual palpation, radiographs, micro-computerized tomography, and histological analysis.

**Results:**

Manual palpation tests showed that the fusion rates of groups III (66.7%), IV (63.6%), V (81.8%), and VI (81.8%) were considerably higher than those of group I. Groups V and VI had higher radiographic scores compared to group I. Micro-CT analysis revealed enhanced bone marrow density expressed as bone volume/tissue volume in groups V (61.88 ± 3.16%) and VI (71.14 ± 3.89%) versus group I (58.26 ± 1.86%). A histological analysis indicated that group VI had enhanced remodeling.

**Conclusion:**

The combination of abundant rhBMP-2 and PTH enhanced bone healing and remodeling of newly formed bone in a rat femoral open fracture model.

## Background

Open fractures of the extremities usually result from high-energy injuries. Because they involve severe soft tissue damage, including stripping of the periosteum around the fracture site, infection and nonunion of open fractures are common [1, 2]. The rates of nonunion and delayed union have been reported as approximately 15% [3], but they can be as high as 45% if vascular injuries occur [4]. When initial treatment is not successful, patients experience prolonged physical, economic, and social disturbances.

Bone morphogenetic proteins (BMPs) are members of the transforming growth factor-β superfamily and powerful osteoinductive molecules. The osteoinductive effects of recombinant human BMPs (rhBMPs) have been documented for fracture repair, the healing of critical bone defects, and spinal fusion in animal models and clinical trials [5–7]. Based on this evidence, rhBMP-2 and rhBMP-7 are approved for clinical use. Nonetheless, results of clinical trials using BMPs have shown that high doses of BMPs are required to induce adequate bone fusion because of the solubility of the molecules, diffusion of the molecules away from the fusion site, and in vivo inactivation [8]. In addition, BMPs are expensive. Therefore, their usefulness is limited. To address these problems, we have tested the osteogenic activity of rhBMP-2 in a rodent model and attempted to generate safer, less expensive, and more efficacious bone fusion using BMPs [9–12].

Human parathyroid hormone (PTH) 1-34 is an anabolic drug, and its efficacy as an osteoporosis drug has been widely verified through experimental and clinical studies [13–16]. Although PTH could have bone resorption effects with continuous administration, it can accelerate bone formation with intermittent administration. PTH can also enhance fracture healing not only in ovariectomized rats but also in intact rats [17, 18]. The clinical use of PTH to accelerate fracture healing has been attempted, but no consistent results have been reported [19].

Although both rhBMP and PTH can enhance fracture healing, the effects of combinations of these drugs have remained largely unknown. The purpose of the present study was to investigate the efficacy and safety of the application of combined BMP and PTH using a rat model of open femoral fractures.

## Methods

### Preparation of matrices

MedGEL (MedGEL, Kyoto, Japan) is a biodegradable gelatin hydrogel scaffolding used for cellular attachment. Although a collagen sponge has been used clinically as a carrier for rhBMP-2 [6, 7], MedGEL has shown more controlled release of rhBMP-2 gradually over 4 weeks and allows longer local retention of rhBMP-2 at the implantation site than a collagen sponge [20], which begins to disintegrate after implantation. The final concentration of rhBMP-2 (Peprotech, Rocky Hill, NJ) was dissolved in phosphate-buffered saline (PBS) buffer (pH 7.5) and applied to MedGEL. MedGEL was cut with a scalpel into 5 × 20-mm strips and placed with rhBMP-2 in an Eppendorf tube that was left overnight at 4 °C prior to implantation. Similarly, 100 μL of rhBMP-2-free PBS was dropped in MedGEL to obtain rhBMP-2-free MedGEL.

### Animals

All animal studies were approved by the Oita University animal research committee, and experiments conformed to all guidelines and regulations for the protection of welfare of animals (protocol No. 1624002).

### Study groups

A total of 72 male Sprague–Dawley rats (8–10 weeks old; CLEA Japan, Inc., Tokyo, Japan) were divided into four groups. Group I (*n* = 12) included animals that were implanted with control carrier alone. Group II (*n* = 12) included animals that were implanted with carrier containing 1 μg of rhBMP-2. Group III (*n* = 12) included animals that were implanted with carrier alone, followed by injections of PTH 1-34 (Forteo; Eli Lilly and Company, Indianapolis, IN). Group IV (*n* = 12) included animals that were implanted with carrier containing 1 μg of rhBMP-2, followed by injections of PTH 1-34. Group V included animals that were implanted with carrier containing 10 μg of rhBMP-2. Group VI included animals that were implanted with carrier containing 10 μg rhBMP-2, followed by injections of PTH 1-34.

### Injections of PTH 1-34

Rats in groups I, II, and V were subcutaneously injected with 0.9% saline solution three times per week beginning 1 week after surgery. Rats in groups III, IV, and VI were subcutaneously injected with PTH 1-34 (20 μg/kg) three times per week (60 μg/kg/week) beginning 1 week after surgery. The injections were continued until immediately before the rats were euthanized.

### Surgical technique used to construct the open femoral fracture model

The Sprague–Dawley rats were anesthetized by an intraperitoneal injection of solution comprising 0.6–0.7 mL of pentobarbital (15 mg/mL) and diazepam (2.5 mg/mL). The left hind limb was prepared for surgery under standard sterile conditions. With the rat in the lateral position, the left femur was located using the posterolateral approach. The periosteum of the femur was circumferentially incised, elevated, and stripped. Then, the femur at the osteotomy site was exposed. A transverse osteotomy was performed at the midshaft of the femoral bone, the fracture fragments were contacted and stabilized, and the intramedullary was fixed using a stainless-steel wire (diameter, 1.0 mm). The wire was cut on the surface of the intercondylar groove to avoid knee joint motion restriction. The material was applied and wrapped around the circumference of the fracture site. The fascial and skin incisions were closed with a 3–0 nylon suture.

Immediately after surgery and on subsequent days, the rodents received analgesics (buprenorphine subcutaneously and paracetamol). The rodents were housed in separate cages and were administered food and water ad libitum; their conditions were monitored daily. The rats were humanely euthanized 8 weeks after surgery, and the operated left femoral bones were explanted and separated from the stainless-steel wire before analysis.

### Manual assessment of fusion

Manual palpation is the most sensitive and specific method of assessing bone fusion [9–12]. After 8 weeks of implantation, the explanted femoral bones were manually tested for intersegmental motion by three blinded independent observers. Any motion detected at either side was considered a failure of fusion. A femoral bone was designated “not fused” if any of the three observers graded it as such. The absence of motion and all-around fusion indicated successful fusion, and such bones were graded as “fused.”

### Radiographic analysis

The explanted femoral bones obtained at the 6–week time point were photographed using a Softex X-ray apparatus (Softex CSM-2; Softex, Tokyo, Japan) using HS Fuji Softex film (Fuji Film, Tokyo, Japan) at 45 cm with 30 kV and 15 mA for 20 s. Fusion was quantified using anteroposterior (A-P) and lateral radiographs. Three blinded independent observers scored the bone formation in each rat using a 4-point scale. Fracture union was judged by visual assessment of the mineralized callus bridging the fracture line on the A-P radiographs (right side: 1 point; left side: 1 point) and lateral radiographs (anterior side: 1 point; posterior side: 1 point).

### Micro-CT analysis

The explanted femoral bones were scanned using SkyScan1172 (Bruker microCT, Kontich, Belgium) with a voxel size of 20 mm. Data were collected at 100 kV and 100 mA and reconstructed using the cone-beam algorithm. Each femoral bone was set on the object stage, and sample scanning was performed over 180° of rotation with an exposure time of 105 ms. A cylindrical volume of interest with a diameter of 20 mm and a height of 27 mm was selected, which displayed the microstructure of the rat femoral bones (comprising the cortical and cancellous bone). Data analysis was performed using CT Analyzer software (Bruker microCT). The region of interest was set at the area of fracture healing and defined by the fracture area filled with new bone; the structural indices of the femoral fracture areas (20 × 20 × 10 mm; a fracture gap in the center) were calculated using this software. During the three-dimensional analysis, tissue volume (TV), bone volume (BV), trabecular thickness (Tb Th), trabecular number (Tb N), and trabecular separation (Tb Sp) were measured.

### Histological analysis

After extraction, the femoral bones were dissected and the specimens were fixed in 40% ethanol. The specimens were then decalcified using a standard 10% decalcifying solution of HCl (Cal-Ex) (Fischer Scientific, Fairlawn, NJ), washed with running tap water, and transferred to 75% ethanol. Serial sagittal sections (5 mm) were carefully cut from the paraffin blocks using a microtome (LS-113; DAIWA-KOKI, Saitama, Japan) at the level of the femoral fracture. They were stained with hematoxylin and eosin and evaluated qualitatively under a light microscope.

### Statistical methods

The Statistical Package for the Social Sciences computer program (SPSS V13; SPSS, Chicago, IL) was used to perform statistical analyses. Fisher’s exact test was used to compare dichotomous variables, and the Kruskal-Wallis test was used for multigroup comparisons of continuous variables; *p* < 0.05 was considered significant. A kappa statistic was calculated as a measure of the interobserver reliability of the three independent blinded observers.

## Results

No abnormal behavior or neurological deficits were noted in any of the 72 rats before or after the surgical procedure or at the time of euthanasia.

### Manual palpation

Table 1 shows the proportion of subjects in each group who achieved fusion according to the three independent evaluators. Consistent agreement (κ = 0.864) was noted among the three independent observers who performed manual palpation. Differences between group I and groups III, IV, V, and VI were statistically significant. A comparison of group II and groups V and VI also resulted in differences in fusion rates.

**Table 1:** Assessment of bony fusion with manual palpation

Treatment group		No. assessed manually for fusion	Fused	Fusion rate (%)
Group I	Carrier alone	11	2	18.2
Group II	1 μg rhBMP-2	12	4	33.3
Group III	Carrier + PTH	12	8	66.7*
Group IV	1 μg rhBMP-2 + PTH	11	7	63.6*
Group V	10 μg rhBMP-2	11	9	81.8*
Group VI	10 μg rhBMP-2 + PTH	11	9	81.8*

*p < 0.05 (vs group I)

### Radiographic analysis

**Fig. 1:**
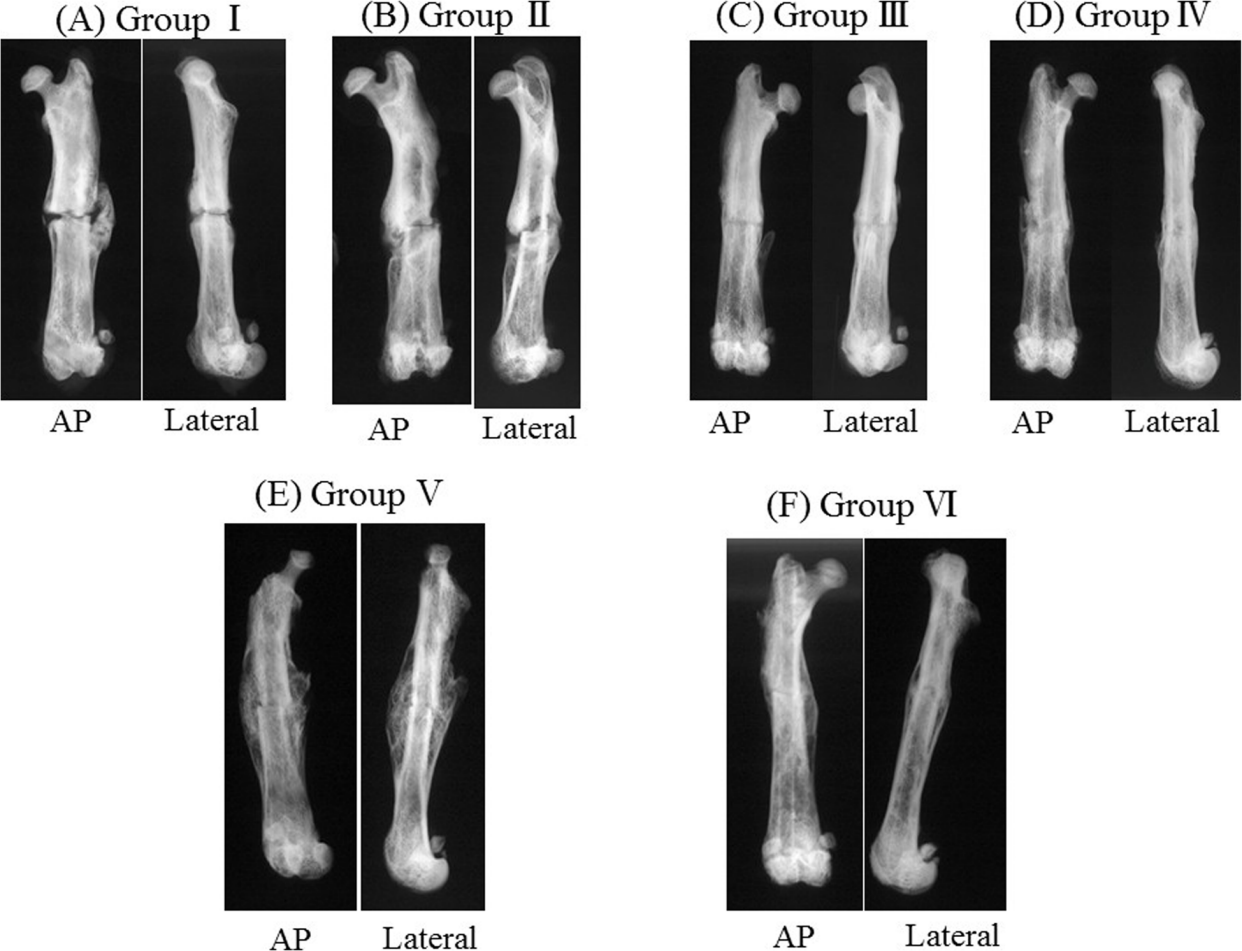
The A-P and lateral radiographs of the representative case in each group at 8 weeks. Fracture lines remained and fractured femurs were not fused in the group I rats (**a**), whereas abundant callus formation was seen at the fracture site of group II (**b**). Rats in groups III (**c**) and IV (**d**) showed bone union of fractured femurs. Rats in group V (**e**) showed bony union of the femur with an expanded callus. Rats in group VI (**f**) showed complete bony union with matured callus

Radiographs of the femurs were obtained at 8 weeks. Consistent agreement (κ = 0.872) was noted among the three independent observers who graded the radiographs. The average evaluation scores for each group are shown in Table 2, and the A-P and lateral radiographs of the representative case in each group at 8 weeks are shown in Fig. 1. Fracture lines remained and fractured femurs were not fused in the group I rats, whereas abundant callus formation was seen in the fracture sites in group II. For rats in groups III and IV, the fractured femurs showed bone union. The femurs of rats in group V showed bony union with an expanded callus. Group VI showed complete bony union with matured callus. The radiographic scores of each group differed statistically (*p* = 0.002) according to the Kruskal-Wallis analysis. A comparison of group I and groups V (*p* = 0.011) and VI (*p* = 0.008) showed significant differences according to the post-hoc analysis.

**Table 2:** Radiographic scores at 8 weeks

Treatment group		No. studied radiographically	Score at 8 weeks (mean ± SD)
Group I	Carrier alone	11	1.18 ± 1.40
Group II	1 μg rhBMP-2	12	1.75 ± 1.42
Group III	Carrier + PTH	12	2.42 ± 1.73
Group IV	1 μg rhBMP-2 + PTH	11	2.55 ± 1.37
Group V	10 μg rhBMP-2	11	3.36 ± 1.21*
Group VI	10 μg rhBMP-2 + PTH	11	3.45 ± 1.04*

*p < 0.05 (vs group I)

### Micro-CT analysis

A computer analysis of the micro-CT images revealed the volume of new bone and the quality of the femoral fracture area. The average micro-CT data based on the histomorphometry of each group are shown in Tables 3 and 4.

**Table 3:** Micro-CT based histomorphometry of femoral fracture at 8 weeks

Treatment group		No. studied micro-CT	TV (mm^3^)	BV (mm^3^)	BV/TV (%)
Group I	Carrier alone	6	607.83 ± 214.20	351.64 ± 111.81	58.26 ± 1.86
Group II	1 μg rhBMP-2	6	528.48 ± 45.28	325.40 ± 24.10	61.68 ± 3.05
Group III	Carrier + PTH	6	473.23 ± 44.46	316.85 ± 18.65	67.23 ± 4.29
Group IV	1 μg rhBMP-2 + PTH	6	483.73 ± 43.25	323.01 ± 36.71	66.74 ± 4.26
Group V	10 μg rhBMP-2	6	480.45 ± 66.31	295.07 ± 34.61	61.88 ± 3.16*
Group VI	10 μg rhBMP-2 + PTH	6	440.52 ± 70.41	313.43 ± 54.46	71.14 ± 3.89*

*TV* tissue volume, *BV* bone volume, BV/TV percent bone volume

*p < 0.05 (vs group 1)

**Table 4:** Micro-CT based histomorphometry of femoral fracture at 8 weeks

Treatment group		No. studied micro-CT	Tb.Th (mm)	Tb.N (1/mm)	Tb.Sp (mm)
Group I	Carrier alone	6	0.63 ± 0.09	0.95 ± 0.13	1.01 ± 0.18
Group II	1 μg rhBMP-2	6	0.67 ± 0.09	0.93 ± 0.10	0.91 ± 0.18
Group III	Carrier + PTH	6	0.77 ± 0.11	0.88 ± 0.10	0.85 ± 0.12
Group IV	1 μg rhBMP-2 + PTH	6	0.71 ± 0.05	0.94 ± 0.03	0.97 ± 0.23
Group V	10 μg rhBMP-2	6	0.60 ± 0.08	1.03 ± 0.04	0.76 ± 0.05
Group VI	10 μg rhBMP-2 + PTH	6	0.70 ± 0.06	1.01 ± 0.08	0.85 ± 0.06

*Tb*.*Th* trabecular thickness, *Tb*.*N* trabecular number, *Tb*.*Sp* trabecular separation

The Kruskal-Wallis analysis revealed a statistical difference (*p* = 0.002) in the variance in the bone volume percentage. The bone volume percentage of group VI was larger than that of group I (*p* = 0.001). Trabecular thickness (*p* = 0.029) and trabecular number (*p* = 0.043) also had statistical significance, whereas post-hoc tests of these two measurements showed no differences in paired comparisons. There were no differences between each group regarding tissue volume, bone volume, or trabecular separation.

### Histological analysis

**Fig. 2:**
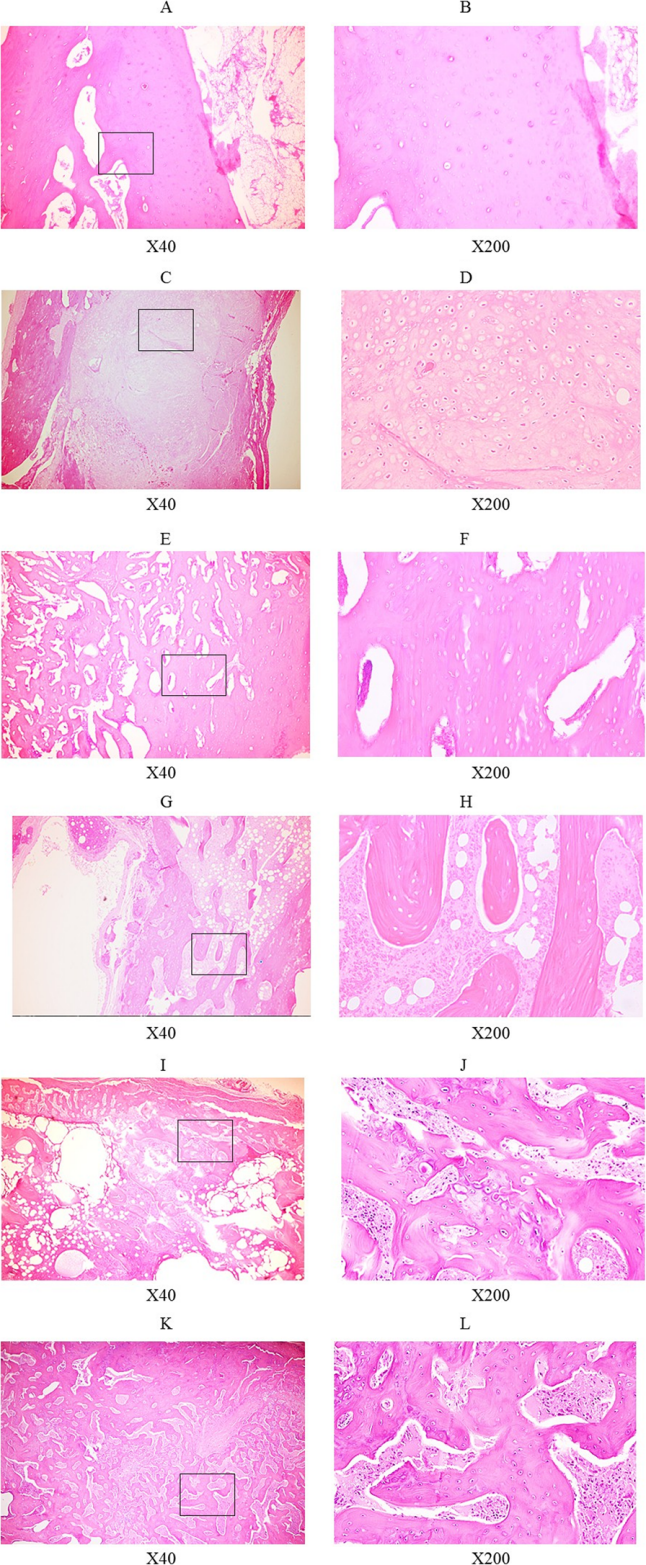
Histological cross-section of the fractured rat femurs obtained 8 weeks after surgery. Group I showed partial cartilaginous tissue formation, but there was no evidence of fusion (**a**, **b**). Group II showed moderate new cartilaginous tissue formation and immature bone formation. Many of the observed chondrocyte cells exhibited expansion (**c**, **d**). Group III showed formation of contracted bone trabeculae. Although general mature bone formation had been observed, a moderate number of chondrocytes remained (**e**, **f**). Group IV exhibited well-developed bone trabeculae and bone marrow. Mature osteocytes were surrounded by bone marrow with increased cavity formation (**g**, **h**). In group V, abundant new bone tissue formed with the expansion of the bone marrow cavity. Although new bone tissue seemed to be mature compared with that of group II, immature chondrocytes remained (**i**, **j**). Group VI exhibited mature new bone formation with finely organized trabecular bone and multinuclear cells indicating osteoclasts, which implied bone remodeling (**k**, **l**)

A microscopic investigation of the groups demonstrated that group I had partial cartilaginous tissue formation, but there was no evidence of fusion (Fig. 2a, b). Group II showed moderate new cartilaginous tissue formation and immature bone formation. Many of the observed chondrocyte cells exhibited expansion (Fig. 2c, d). Group III showed formation of contracted bone trabeculae. Although mature bone formation had been seen, a moderate number of chondrocytes remained (Fig. 2e, f). Group IV showed well-developed bone trabeculae and bone marrow. Mature osteocytes were surrounded by bone marrow with increased cavity formation (Fig. 2g, h). In group V, abundant new bone tissue was formed with the expansion of the bone marrow cavity. Although new bone tissue seemed to be mature compared with that of group II, immature chondrocytes remained (Fig. 2i, j). Group VI indicated mature new bone formation with finely organized trabecular bone and multinuclear cells indicating osteoclasts, which implied bone remodeling (Fig. 2k, l).

## Discussion

We investigated the effects of a combination of locally applied rhBMP-2 and intermittent administration of PTH after surgical intervention to enhance fracture repair in a well-accepted rodent model of open femoral fractures. According to our previous studies [9–12], our method of surgical intervention, which stripped the periosteum of the femur before cutting the bone, induced prolonged bone healing in a rat model of femoral fractures. We used MedGEL as a carrier to allow more controlled and sustained release of the rhBMP-2 so that the growth factor concentration was maintained locally within the therapeutic range to gain the maximum therapeutic effect [20]. Results of manual palpation and radiographic assessment tended to enhance bone healing in group II compared with group I, and group V showed significantly enhanced bone healing during manual palpation, radiographic assessment, and bone volume fraction (BV/TV) during micro-CT analysis. The results were comparable with our previous findings [11, 12] indicating that the application of 1 μg of rhBMP-2 with carrier effectively enhanced bone healing in a rat model of femoral fractures, although it was not enough to achieve complete bone union; 10 μg of rhBMP-2 was necessary for complete bone union. BMP-2 is available for use in the clinical setting, but its expense is problematic. A low dosage of BMP-2 is ideal for reducing costs and avoiding adverse effects associated with a high dosage of rhBMP-2 [21]; however, a high dosage of BMP-2 might be necessary for impaired healing of open fractures. In the present study, group V had a better fusion rate than group II according to the manual tests. The histological analysis revealed markedly enhanced callus formation with the expansion of the bone marrow cavity in group V.

Regarding the effectiveness of PTH, PTH-treated group III had significantly better fusion rates than group I according to manual palpation testing. Group III also tended to have higher radiographic scores compared with group I. The results of attempting to enhance bone healing with intermittent administration of PTH were not surprising because several previous studies reported that PTH induced callus formation and enhanced bone union in both ovariectomized rats and intact rats [13–16]. Although a generous amount of PTH effectively demonstrated fracture healing in rat models [17], the clinical application for humans must be much larger. A clinical dose of PTH to treat human osteoporosis is 20–40 μg daily in Japan and the USA. A dose of 5 μg/kg/day seemed ineffective for changing the mechanical properties in a rat model of fractures [18]. Nakajima et al. reported successful enhancement of the callus mechanical strength with daily doses of 10 μg/kg/day PTH in intact rat femurs [16]. Therefore, we adopted 20 μg/kg alternate-day dosing (60 μg/kg/week) because daily dosing and alternate-day dosing did not have an observable impact on the results [22].

Recent studies of the interactions between PTH and BMP showed that there are two types of interactions between PTH and rhBMP-2 [23]. The first is a mechanism that occurs through a complex comprising PTH type-1 receptors (PTH1R), low-density lipoprotein receptor-related protein 6 (LRP6) within the canonical Wnt pathway, and BMP antagonists. Endocytosis of the complex suppresses negative feedback of BMP antagonists, thereby stimulating BMP signaling. Through that mechanism, PTH enhances the differentiation of mesenchymal stem cells (MSCs) into osteoblasts. The second is a mechanism that occurs through peroxisome proliferator-activated receptor gamma (PPARγ). Excessive BMP signaling results in downregulation of the Wnt pathway through sclerotin. The downregulated Wnt pathway results in increased PPARγ, which persuades MSCs to differentiate into adipocytes. PTH could activate the Wnt pathway and suppress PPARγ. Morimoto et al. reported synergistic anabolic effects with the concomitant use of intermittent PTH and locally applied rhBMP-2 in a rat model of spinal fusion [22]; in their study, the combination increased fusion rates and improved the quality of the newly formed bone. In the present study, groups IV and VI had significantly better fusion rates than group I according to the manual palpation tests, and group VI had higher radiographic evaluation scores. Group VI also had a better fusion rate than group II. The micro-CT analysis indicated that group VI had significantly higher BV/TV scores compared to group I, which indicated increased bone marrow density. Trabecular thickness (Tb.Th) and trabecular number (Tb.N) also showed statistical differences, whereas a paired comparison showed that Tb.Th and Tb.N had no significance according to post-hoc tests.

To interpret these results under the circumstance of impaired bone healing of an open fracture, PTH could be partially effective for enhancing bone healing; however, a slight amount of adjunctive BMP-2 did not seem to have a synergistic effect. An abundant amount of BMP-2, which stimulates callus formation, is necessary for PTH to elicit its enhanced bone healing effects. Taegil et al. [24] reported that the open fracture group treated with PTH had a bony fusion rate similar to that of the open fracture group treated with saline. This study implied that some stimulators such as rhBMP-2 would be necessary for open fractures in which bone anabolism is attenuated. Although we could not statistically prove any additive or synergistic effects with the combination of rhBMP-2 and PTH, we presumed that their combination could promote remodeling in a rat model of open femoral fractures. The results of the histological analysis revealed that group IV had well-developed bone trabeculae and bone marrow and mature osteocytes that were not seen in groups II and III, and group VI showed an almost normal appearance of the bone marrow compared to group V, in which the fusion rate and bone marrow density were similar to those of group VI. This could be evidence of enhanced remodeling caused by the combination of rhBMP-2 and PTH.

Limitations of the present study was that we could not see the healing process of the femur because we decided to sacrifice the rats at 8 weeks after surgery, when complete remodeling of the fractured bone had occurred. Despite this, we chose to use a practical dose of rhBMP-2 and PTH in the clinical setting, which was advantageous to the study. Although we cannot directly apply our findings to humans because the rat has different biological reactions to drugs, we believe that the results of the current study provide further evidence for understanding the effects of these therapeutic agents.

## Conclusion

The present study demonstrated the effects of using a combination of rhBMP-2 and intermittent PTH for bone fracture repair in a rat model of open femoral fractures. PTH showed a better fusion rate according to the manual palpation test, but rhBMP-2 is the key to enhancing bone healing of open fractures. Therefore, a combination of abundant rhBMP-2 and PTH could enhance bone healing and remodeling of newly formed bone in a rat femoral open femoral model.

## Data Availability

All data generated or analyzed during this study are included in this published article.

## Abbreviations

BMP-2: Bone morphogenetic protein 2
BMPs: Bone morphogenetic proteins
BV: Bone volume
LRP6: Lipoprotein receptor-related protein 6
PBS: Phosphate-buffered saline
PPARγ: Peroxisome proliferator-activated receptor gamma
PTH: Parathyroid hormone
PTH1R: PTH type-1 receptors
rhBMPs: Recombinant human BMPs
Tb.N: Trabecular number
Tb.Th: Trabecular thickness
TV: Tissue volume
